# Comparison of the Left Main Coronary Bifurcating Angle among Patients with Normal, Non-significantly and Significantly Stenosed Left Coronary Arteries

**DOI:** 10.1038/s41598-017-01679-3

**Published:** 2017-05-04

**Authors:** Yu-Hsiang Juan, Pei-Kwei Tsay, Wei-Chih Shen, Chih-Seng Yeh, Ming-Shien Wen, Yung-Liang Wan

**Affiliations:** 1 Department of Medical Imaging and Intervention, Chang Gung Memorial Hospital at Linkou, Institute for Radiological Research, College of Medicine, Chang Gung University, (333) Taoyuan, Taiwan; 2 Department of Public Health and Center of Biostatistics, College of Medicine, Chang Gung University, (333) Taoyuan, Taiwan; 3 Department of Medical Research, China Medical University Hospital, China Medical University, (40447) Taichung, Taiwan; 4 Section of Cardiology, Department of Internal Medicine, Chang Gung Memorial Hospital at Linkou, College of Medicine, Chang Gung University, (333) Taoyuan, Taiwan

## Abstract

We evaluated the correlation of the left main coronary bifurcating angle (LCBA) with the severity of coronary atherosclerosis, risk factors of coronary artery disease (CAD) and the feasibility of measuring the LBCA using the axial plane. Coronary Computed tomography angiographies (CTAs) of 313 patients between Nov. 2006 and Oct. 2013 were reviewed and separated into three groups. Group I (211 patients) had significant stenosis (≥50%) of the left anterior descending coronary artery (LAD) and/or left circumflex coronary artery (LCX). Group II (62 subjects) had atherosclerosis without significant stenosis. Group III (40 subjects) had unremarkable coronary CTAs. Both Group I and II patients received conventional catheter angiography to confirm the severities of coronary stenoses. Significant differences were found among the groups with respect to risk factors, such as male gender, hypertension and body mass index. Axial plane measurement was feasible in most patients (82.1%), without significant differences among the groups. The mean LCBA was 84.7° among all patients, and significantly differed among groups I, II and III (87.34°, 81.16° and 75.53°, P < 0.001). The LCBA of group I was significantly higher than group III (P < 0.001) in univariate analysis, but insignificant in multivariate analysis (P = 0.064).

## Introduction

During the past decade, coronary computed tomography angiography (coronary CTA) has been accepted as a highly reliable and less invasive modality for CAD diagnosis owing to its high spatial and temporal resolution and excellent diagnostic accuracy^1–5^. Coronary CTA allows the visualization of coronary artery anatomy and the associated atherosclerotic plaques^6–8^. This is represented in the ability of coronary CTA to identify the coronary anatomy as well as the location, distribution, characteristics and composition of plaques in the coronary arteries^6, 7, 9^.

The evaluation of the left main coronary bifurcation angle (LCBA) has gained increasing clinical concern and research interest because the angulation of the LCBA has been shown to have a hemodynamic effect on shear stress, flow turbulence formation and the consequent development of plaques at the bifurcating regions; thus, measurements of the bifurcation angles will provide an insight into demonstrating the relationship among the plaques, CAD and coronary angles^4–6, 8, 10–17^. In previous studies, a wider bifurcation angle has been hypothesized as related to higher turbulence and low shear stress, which might induce plaque proliferation at the bifurcated regions, whereas a narrow angle might be more prone to present laminar flow and less likely to induce plaque formation^4–6, 8, 10–18^. Despite the evidence of a direct correlation between the LCBA and the formation of plaques^4^, to the best of the authors’ knowledge, this is the first study to investigate the correlation between the LCBA and the development of non-significant atherosclerosis and significant stenosis in the left anterior descending artery (LAD) or left circumflex artery (LCX). In addition, we would like to assess the feasibility of measuring the LCBA from axial slices alone, which can potentially be measured directly without the need for additional imaging reformation.

## Materials and Methods

### Patient Selection and Demographic Data

This retrospective study was approved by the institutional review board (Chang Gung Medical Foundation), and informed consent for all the coronary CTAs and CCAs was obtained from all subjects. The data were collected from the previous coronary CTAs that were performed between November 2006 and October 2013. In total, 313 patients who had optimal coronary CT angiographs with an acceptable image quality to assess the LCBA were enrolled in this retrospective study. The ages of the 313 patients ranged from 35 to 84 (57.7 ± 10.2) years, and 258 were male.

The patients were separated into three groups depending on whether there was significant stenosis or atherosclerotic plaques of either the LAD or LCX. Group I comprised patients who had significant stenosis (≥50% reduction in the luminal diameter, 211 patients) of the LAD and/or LCX. Group II comprised 62 subjects who had atherosclerosis of the LAD and/or LCX, but without significant stenosis. The patients were allocated to either group I or group II after examination by conventional invasive coronary angiography. Group III comprised 40 subjects for whom the CTAs were unremarkable and the calcium scores were zero.

The demographic and essential data of each patient were collected and recorded; these included age; gender; body height; body weight; body mass index (BMI); systolic and diastolic blood pressure; risk factors of CAD, including hypertension, obesity, diabetes mellitus, smoking history, and hypercholesterolemia; and heart rate during scanning. The demographic data and risk factors of the patients in the 3 groups are listed in Table 1.

**Table 1:** Demographic data and risk factors of the subjects in the three groups.

	Group I (n = 211)	Group II (n = 62)	Group III (n = 40)	P-value^*^
Age (Mean ± SD)	58.14 ± 10.34	58.24 ± 10.01	54.70 ± 9.62	0.135
Gender (male)	188 (89.1%)	50 (80.6%)	20 (50.0%)	<0.001*
BH (cm)	165.18 ± 7.06	163.86 ± 8.98	163.38 ± 9.21	0.258
BW (kg)	73.50 ± 11.71	70.63 ± 12.22	70.61 ± 19.86	0.193
BMI (kg/m^2^)	26.92 ± 3.66	26.21 ± 3.47	25.49 ± 3.58	0.047*
SBP (mm Hg)	136.51 ± 21.74	134.56 ± 20.14	131.93 ± 20.17	0.423
DBP (mm Hg)	79.89 ± 11.65	81.81 ± 11.26	85.08 ± 9.71	0.025*
HR (per minute)	61.36 ± 8.36	62.46 ± 6.65	63.40 ± 7.55	0.265
Diabetes Mellitus	54 (25.6%)	8 (12.9%)	5 (12.5%)	0.034*
Hypertension	110 (52.1%)	37 (59.7%)	13 (32.5%)	0.024*
Smoking	25 (11.8%)	11 (17.7%)	3 (7.5%)	0.278
Obesity	104 (49.3%)	21 (33.9%)	8 (20.0%)	<0.001*
Hypercholesterolemia	48 (22.7%)	12 (19.4%)	7 (17.5%)	0.689
Feasibility of Axial measurement	171 (81.0%)	52 (83.9%)	34 (85.0%)	0.905
Presence of Ramus intermedius	115 (54.5%)	29 (46.8%)	20 (50.0%)	0.543

Group I = presence of significant stenosis of either the left anterior descending artery, the left circumflex artery or both. Group II = presence of plaques in either the left anterior descending artery, the left circumflex artery or both, but without associated significant stenosis, Group III = Normal subjects who had negative findings on coronary CT angiography, with a zero calcium score. BW = body weight; BH = body height; BMI = body mass index; SBP = systolic blood pressure; DBP = diastolic blood pressure; LCBA = visualization of the left coronary bifurcation within 1 to 3 axial CT images. *Denotes statistical significance, with a p value < 0.05.

### Patient Preparation for Coronary CTA

Patients were given 5 to 45 mg of the β-blocker propranolol (10 mg in each tablet, AstraZeneca UK, Limited, Cheshire, United Kingdom) 30 minutes to one hour before the scan if their heart rate (HR) was ≧65 beats per minute (bpm). Alternatively, esmolol (esmolol HCL, 10 mg/mL, JenYa Biotech Incorated., Hsing Chu, Taiwan) was administered intravenously at a dosage of 0.5 mg/Kg under electrocardiographic monitoring if there were contraindications for administering the β-blocker Inderal (such as asthma, AV conduction block, and Raynaud syndrome) or if the heart rate was persistently greater than 65 bpm after Inderal was administered. A dosage of 0.3 to 0.6 mg of nitroglycerin (Pfizer Pharmaceuticals, LLC, Puerto Rico, USA) was administered sublingually 3 minutes prior to scanning to improve the coronary artery imaging.

### Technique for CTA and Coronary calcium scoring

All patients underwent Agatston coronary calcium scoring and subsequent coronary CTA using either a 320-detector CT scanner (Aquilion ONE, Toshiba Medical Systems, Otawara, Japan) or a 64-detector CT machine (Aquilion 64, Toshiba Medical Systems, Tokyo, Japan). The former scanner had 320 rows of detectors, each 0.5 mm wide, and a gantry rotation time of 0.35 s with craniocaudal or z-axial coverage of up to 160 mm. The later scanner had a detector collimation of 64 × 0.5 mm, and a gantry rotation time of 0.40 ms with a z-axis coverage of 32 mm. Tube voltages (kVs) and currents (mAs) were selected according to the manufacturer’s suggestions from the patients’ body mass indices.

The 320-detector CTAs were performed under prospectively ECG-triggered axial acquisition according to previous published criteria^19^. If a patient’s heart rate was ≦65 bpm, then a one-heartbeat acquisition with half-scan reconstruction was performed at the mid-diastolic phase (65–85%). For heart rates ranging from 66 to 79 bpm and ≧80 bpm, the patients were scanned with either two- or three-heartbeat acquisitions, along with a wider phase window (35–85%), if necessary. For vascular enhancement, a bolus of contrast medium (Iohexol; Omnipaque 350, Amersham Health Co., Cork or Ireland, GE Healthcare) was administered intravenously according to the patients’ body mass indices (60 mL for body mass index ≦28 and 70 mL for those with a body mass index >28) at a rate of 5 mL/sec through the antecubital vein via a power injector followed by 40 mL of a saline flush. CT scanning was started manually after visible contrast medium opacification of the left ventricle.

The 64-detector CT angiography was also performed in accordance with the prior suggested criteria^20^. Retrospective ECG gating was employed with the timing bolus method. The scanning parameters were as follows: gantry rotation time of 350–500 ms with heart rate–adjustment, tube voltage of 120 kV, and pitch and tube currents determined based on the patients’ weights, as previously published. For vascular enhancement, a bolus of contrast material with either iodixanol (Visipaque 320 mg I/ml, Amersham Health, Buckinghamshire, UK) or iohexol (Omnipaque 350, Amersham Health Co., Cork or Ireland, GE Healthcare) was administered intravenously through the antecubital vein at a rate 4–5 ml/s and a total volume between 80–100 ml, followed by 50 mL of saline chasing at 3.5 mL/sec using a power injector.

### CCA Technique

CCA was performed by experienced cardiologists using either a Philips (Integris BH3000, Philips Healthcare, Best, The Netherlands) or a Siemens (AXIOM-Artis, Siemens, Forchheim, Germany) angiographic machine. All group I and II patients had CCA within 90 days (for subjects who underwent 64-detector CTA) or within 4 days (for subjects who underwent 320-detector CTA). As with the coronary CTA, the CCA was also performed according to a previously published method^19^. Aspirin (300 mg) and clopidogrel (300 mg) were given orally before angiography. The procedure was performed according to standard Seldinger techniques on an angiographic machine using either the femoral or the radial artery as the arterial access. Images were recorded at 15 frames per second at a resolution of 512 × 512 pixels for analysis. CCA was performed via the true left anterior oblique (LAO), right anterior oblique (RAO), and RAO caudal views, while the LAO cranial view was used for both the LCX and the LAD and the right anterior oblique view for the RCA. The severity of vascular stenosis on the angiograms was analyzed using a quantitative analysis software package (Scientific QCA Analysis, Siemens, Germany) by cardiologists blinded to the coronary CTA data. The minimal lumen diameter was measured in projections showing the most severe narrowing. As mentioned previously, the patients were classified as group II if the coronary stenosis was insignificant (defined as luminal diameter reduction <50% on QCA and as group I if the coronary stenosis was significant (defined as luminal diameter reduction ≥50%).

### Image Analysis and Measurement of the Left Coronary Bifurcation Angle

**Figure 1:**
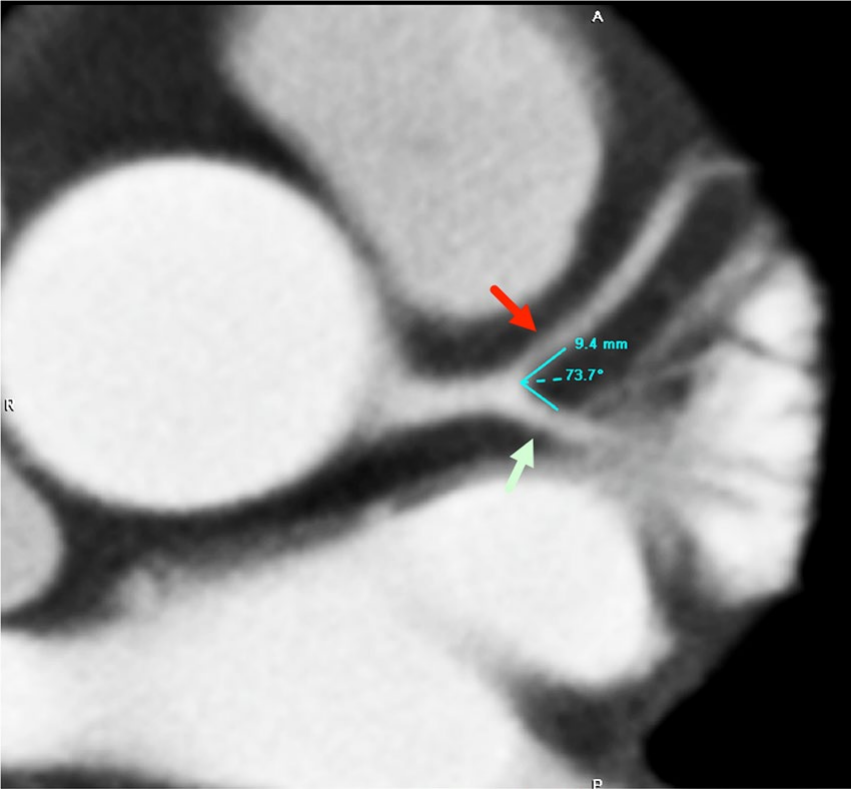
A 53-year-old male with normal findings of the LAD (red arrow) and LCX (light green arrow). The calcium score was zero. The angle between the LAD and the LCX measures 73.7°.

**Figure 2:**
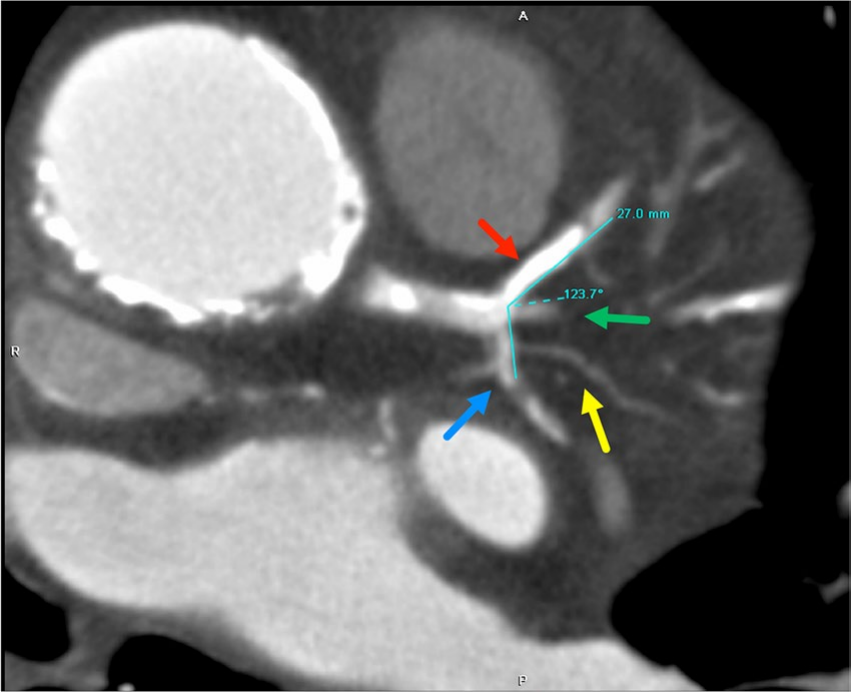
A 73-year-old male who had significant stenosis of the LAD (red arrow) and the LCX (dark blue arrow). The calcium score of the LAD was 670. The total calcium score was 3775. The angle between the LAD and the LCX (dark blue arrow) was 123.7°. The ramus intermedius (green arrow) and obtuse marginal branch 1 (yellow arrow) are also obvious in the axial image.

The DICOM data (digital imaging and communication in medicine) obtained from the PACS (picture archiving and communicating system) were transferred to a workstation equipped with validated commercial software (Vitrea, Vital Images, Inc., MN, USA) for generating multiplanar formation, curved planar reformation, and 3-dimensional volume rendering images along with the axial images for assessing the LCBA between the LAD and LCX (Figs 1 and 2). We also recorded the feasibility of measuring the LCBA using 1–3 axial images alone (Fig. 2). Since the study was retrospective in nature, all the LCBA measurements were performed according to the best image phase and were not limited to the diastolic phase only, which was also in accordance with the real-world clinical situation. The LCBA measurement from axial images was considered feasible if it could be easily visualized and measured using 1–3 adjacent axial images alone. The recorded LCBA was obtained using a combination of axial, multiplanar reformation and/or 3-dimensional volume rendering images. The decision was based on the easiest and best-visualized imaging method and depended on the judgment of the radiologist performing the measurement.

### Statistical analysis

All quantitative variables are expressed as the mean ± standard deviation. Categorical variables are expressed as numbers and percentages. The continuous variables and differences among the groups were obtained using an ANOVA test with multiple comparisons employing the Bonferroni method. For the categorical data, the P-value was calculated by the Chi-square test. Multivariate analysis was performed using logistic regression. A p value less than 0.05 was considered significant. The statistical analysis was performed using SPSS software (version 17; SPSS, Inc., Chicago, Illinois, USA).

## Results

**Figure 3:**
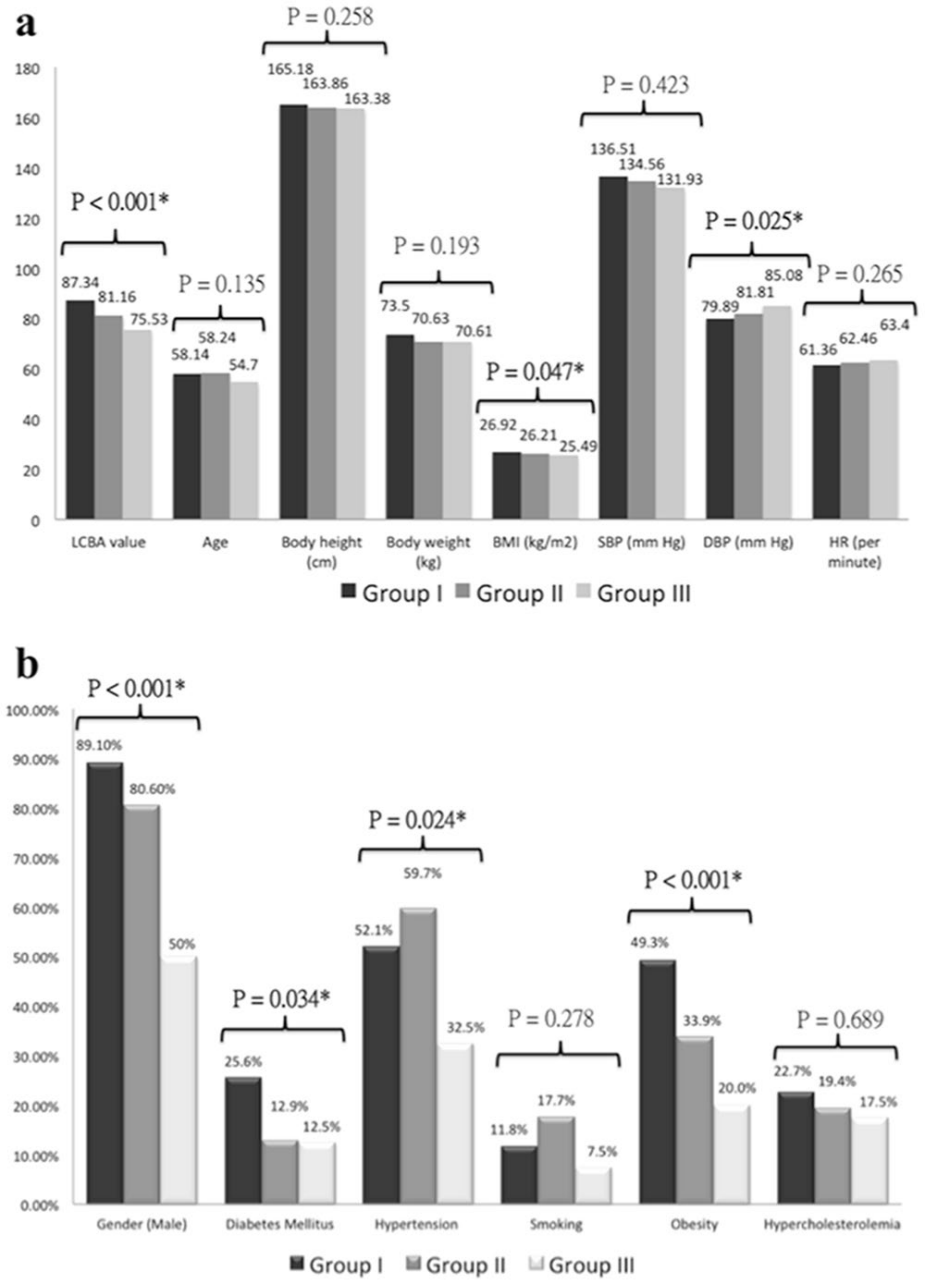
Comparison of the conventional risk factors for coronary artery disease and the LCBA values among the three groups of patients with respect to (**a**) continuous variables and (**b**) categorical variables. BMI = body mass index, SBP = systolic blood pressure, DBP = diastolic blood pressure, HR = heart rate. The asterisk (*) denotes statistical significance.

The differences in demographic data and risk factors are summarized in both Table 1 and Fig. 3. These groups (groups I, II and III) showed significant differences with respect to male gender (89.1% vs 80.6% vs 50%, P < 0.001), diabetes mellitus (25.6% vs 12.9% vs 12.5%, P = 0.034), obesity (49.3% vs 33.9% vs 20%, P < 0.001), hypertension (52.1% vs 59.7% vs 32.5%, P = 0.024) and BMI (26.92 ± 3.66 vs 26.21 ± 3.47 vs 25.49 ± 3.58, P = 0.047). By contrast, group I had a lower diastolic blood pressure than did the other two groups (79.89 ± 11.65 vs 81.81 ± 11.26 and 85.08 ± 9.71, P = 0.025). There were no significant differences in the other demographic data, risk factors and presence of ramus intermedius. Overall, the LCBA could be visualized and assessed within 1–3 axial image planes (Figs 1 and 2) in most patients, including 82.1% overall (313 subjects), 81.0% in group I, 83.9% in group II and 85% in group III; the figures for these 3 groups were not significantly different (P = 0.905).

Figure 3 summarizes the comparison of the LCBA values among the 3 groups. The mean LCBA was 84.7° ± 19.0 (range, 35.8 to 141.8 degrees) among all 313 patients (Figs 1 and 2). Overall, the LCBA showed significant differences among the three groups (87.34° ± 18.84 vs 81.16° ± 20.06 vs 75.53° ± 14.71, P < 0.001). Group I (Fig. 2) had a significantly higher LCBA compared with group III (87.34° ± 18.84 vs 75.53° ± 14.7, P < 0.001) (Fig. 1). The LCBA of group I was higher than that of group II (87.34° ± 18.84 vs 81.16° ± 20.06), and the LCBA of group II was higher than that of group III (81.16° ± 20.06 vs 75.53° ± 14.7), but neither of these differences were statistically significant (P = 0.117 and 0.313, respectively).

We have performed additional multivariate analysis on the significant primary endpoints. Since multicollinearity exists among hypertension, diastolic blood pressure, obesity and BMI, we choose one of these (obesity) as a representative for analysis. We found that gender and obesity remained significant when comparing group I and group III patients (P < 0.001 and 0.022, respectively), while gender and hypertension were significantly different when comparing group II and group III patients (P = 0.004 and 0.022, respectively). Although the left main coronary bifurcating angle is significant in univariate analysis, it becomes insignificant, though close to reaching statistical significant, in multivariate analysis (P = 0.064). The results from the multivariate analysis were summarized as Table 2.

**Table 2:** Multivariate analysis of demographic data, risk factors and left main coronary bifurcating angle.

	Odds ratio	Odds ratio 95%CI	P-value*
**Comparison of group I and group III**	**Odds ratio**	**Odds ratio 95%CI**	P-value*
Left Main Coronary Bifurcating Angle	1.02	1.00–1.05	0.064
Gender (male)	7.94	3.35–18.81	**<0**.001*
Diabetes Mellitus	2.38	0.81–7.05	0.116
Hypertension	2.08	0.91–4.78	0.085
Obesity^#^	2.98	1.17–7.53	**0**.022*
**Comparison of group II and group III**	**Odds ratio**	**Odds ratio 95%CI**	P-value*
Left Main Coronary Bifurcating Angle	1.01	0.99–1.04	0.346
Gender (male)	4.52	1.63–12.54	**0**.004*
Diabetes Mellitus	0.76	0.20–2.92	0.687
Hypertension	3.08	1.17–8.08	**0**.022*
Obesity^#^	1.96	0.65–5.85	0.231

Group I = presence of significant stenosis of either the left anterior descending artery, the left circumflex artery or both. Group II = presence of plaques in either the left anterior descending artery, the left circumflex artery or both, but without associated significant stenosis, Group III = Normal subjects who had negative findings on coronary CT angiography, with a zero calcium score. *Denotes statistical significance, with a p value < 0.05. ^**#**^Since multicollinearity exists among hypertension, diastolic blood pressure, obesity and body mass index, we choose one of these (obesity) as a representative for analysis.

## Discussion

A wider LCBA has been shown to produce a region of low wall shear stress in the left main bifurcating regions, which has drawn much research interest regarding the effect of LCBA on the development of CAD^4, 5, 8, 10, 12, 13, 17, 18^. A prior study using intravascular ultrasonography confirmed the higher prevalence of atherosclerotic plaques in the left main bifurcation area rather than in the left main coronary artery^4, 21, 22^, which also established the basis for the concept of using the LCBA to assess the left coronary bifurcation. To further expand our knowledge regarding LCBA and CAD development, our study not only evaluated the clinical application of the LCBA in a group with significant coronary stenosis (group I) but also compared this group with patients presenting atherosclerosis but no significant stenosis (group II) and with patients presenting normal coronary arteries (group III). We believe there is a clinical interest in understanding the trend of LCBA change according to the severity of atherosclerosis, but, to the best of our knowledge, such a comparison among three groups of patients has not been performed before.

Prior literatures emphasized the association of a wider LCBA with the development of CAD and a cut-off value of 80 degrees for predicting the presence of CAD^4, 5, 11, 15, 17^. The results of our study corresponded with a cut-off value of greater than 80 degrees for detecting CAD (mean LCBA 87.34° for group I). Significant differences were noted in the LCBA values among the three groups, with group I significantly higher than group III from univariate analysis (P < 0.001). However, the difference is insignificant in multivariate analysis, though very close to reaching a statistical significance (P = 0.064). We propose that the possible reasons of the statistical insignificance may be the co-influence of LBCA with the risk factors, and also the relative lower case number of group III as compared to group I. The mean angle measured in patients with a diseased LAD has been reported to be 94° ± 19.7^4^ and 80° ± 27^15^, whereas the corresponding figure was 87.34° ± 18.84 in the group I patients and 81.16° ± 20.06 in the group II patients. Our measured values correlate well with the average bifurcation angle reported by prior studies. In a series of patients with LAD disease, 15 (68%) had a bifurcation angle >80 degrees, and 89% of the patients with both LAD and LCX disease had a bifurcation angle >90 degrees^4^.

Although the LCBA values of group I compared with group II and of group II compared with group III were both higher, they did not reach significantly higher levels compared with group III, as we initially expected (P = 0.117 and 0.313, respectively). We believe this is mainly because our study was performed at a single medical center with limited number of patients. Secondly, it is noteworthy that the mean LCBA in our normal subjects in group III was 75.5° ± 14.7, which is nearly identical to the value of 75.5° ± 19.8 reported by Sun *et al*.^4^. We also evaluated for the presence of ramus intermedius, which showed no significant difference among the three groups of patients. Previous studies have reported a varying prevalence of ramus intermedius, ranging from approximately 15–31%^23–25^, whereas our study showed a higher prevalence of ramus intermedius, up to approximately 45–50%. The difference may be due to the study cohort or due to the inclusion of all of the visible cases of ramus intermedius in our study.

A recent investigation by Temov *et al*. showed a significant association of male gender and BMI with CAD development^5^. Our study takes a further step in a comparison of three groups of patients and revealed a significant difference among the three groups in terms of the following risk factors: male gender (P < 0.001), diabetes mellitus (P = 0.034), obesity (P < 0.001), hypertension (P = 0.024) and BMI (P = 0.047), for which the male gender and BMI are in accordance with the results of Temov *et al*.^5^. Subsequent multivariate analysis showed that gender and obesity remained significant when comparing group I and group III patients (P < 0.001 and 0.022, respectively), while gender and hypertension were significantly different when comparing group II and group III patients (P = 0.004 and 0.022, respectively). Male sex, BMI and obesity may have an influence on the body habitus of the patient, which subsequently expands the LCBA, whereas hypertension may play a role through left ventricular hypertrophy. As mentioned above, we believed that LCBA may have co-influence with the clinical risk factors. Further studies focusing on the long-term follow-up of systolic and diastolic blood pressures may help to better understand their influence on the LCBA and CAD development.

Our study disclosed that the LCBA could be well visualized and assessed within one to three axial scanning planes; the frequency of such assessments was 82.1% in the total 313 subjects, 81.0% in group I, 83.9% in group II and 85% in group III, without significant differences among these three groups (P = 0.905). We believe that, to assess the LCBA alone, an initial attempt using the axial plane may be possible because approximately 82% of patients overall are approachable by axial images alone, which can minimize the necessity for time-consuming multiplanar and 3-dimensional volume-rendering reformations. To the best of our knowledge, this is also the first study to report the feasibility of LCBA measurements using the axial plane.

There are several limitations to this study. First, the diameters of the LM, LAD and LCX were not investigated for the correlation between the arterial diameter and atherosclerosis. However, the presence of significant and non-significant stenosis of the LAD and LCX should be a more critical issue than the arterial diameter. Second, the LCBA values on angiograms were not measured for comparison; however, since CCA had been shown to have limitations (such as foreshortening and out-of-plane magnification), coronary CTA has been demonstrated to be an accurate tool to measure the LCBA^14, 17^. Three-dimensional quantitative coronary angiography may help to resolve this problem, but the availability is limited. Third, the 40 normal subjects in group III did not undergo CCA for confirmation; however, this could be compensated by the fact that the coronary CTA has a negative predictive rate of nearly 99%^19^. Fourth, since we believed that the hemodynamic effect of a widening LCBA might have an influence beyond the bifurcating region, we did not limit the study to patients with plaques and stenosis occurring only in the bifurcating region. The presence of heart failure or cardiomegaly may be a contributing factor for LCBA widening; however, we did not perform an additional subgroup analysis since the purpose of this study was to understand the effect of the LCBA on the general clinical patients in the three groups of subjects. Fifth, although the LCBA was measured through an offline analysis of coronary CTA, it was not possible to blind those assessing the LCBA to the presence or absence of obstructive CAD by coronary CTA. Sixth, there was likely a significant variation in the quality of the coronary CTA scans given that a proportion were performed using a 320-detector scanner with prospective gating and the remainder using a 64-detector scanner with retrospective ECG-gating. Lastly, our study had a relatively less number of patients in group III as compared to the other groups.

## References

[1] Raff GL, Gallagher MJ, O′Neill WW, Goldstein JA (2005). Diagnostic accuracy of noninvasive coronary angiography using 64-slice spiral computed tomography. J Am Coll Cardiol.

[2] Rybicki FJ (2008). Initial evaluation of coronary images from 320-detector row computed tomography. Int J Cardiovasc Imaging.

[3] Schuijf JD (2006). Diagnostic accuracy of 64-slice multislice computed tomography in the noninvasive evaluation of significant coronary artery disease. Am J Cardiol.

[4] Sun Z, Cao Y (2011). Multislice CT angiography assessment of left coronary artery: correlation between bifurcation angle and dimensions and development of coronary artery disease. Eur J Radiol.

[5] Temov K, Sun Z (2016). Coronary computed tomography angiography investigation of the association between left main coronary artery bifurcation angle and risk factors of coronary artery disease. Int J Cardiovasc Imaging.

[6] Cademartiri, F. *et al*. Diagnostic accuracy of multislice computed tomography coronary angiography is improved at low heart rates. *Int J Cardiovasc Imaging* **22**, 101–105, doi:10.1007/s10554-005-9010-6; discussion 107–109 (2006). 10.1007/s10554-005-9010-6 16077999

[7] Mollet NR (2005). High-resolution spiral computed tomography coronary angiography in patients referred for diagnostic conventional coronary angiography. Circulation.

[8] Rodriguez-Granillo GA, Rosales MA, Degrossi E, Durbano I, Rodriguez AE (2007). Multislice CT coronary angiography for the detection of burden, morphology and distribution of atherosclerotic plaques in the left main bifurcation. Int J Cardiovasc Imaging.

[9] Schuijf JD (2007). Differences in plaque composition and distribution in stable coronary artery disease versus acute coronary syndromes; non-invasive evaluation with multi-slice computed tomography. Acute Card Care.

[10] Chaichana T, Sun Z, Jewkes J (2011). Computation of hemodynamics in the left coronary artery with variable angulations. J Biomech.

[11] Girasis C (2010). 3-Dimensional bifurcation angle analysis in patients with left main disease: a substudy of the SYNTAX trial (SYNergy Between Percutaneous Coronary Intervention with TAXus and Cardiac Surgery). JACC Cardiovasc Interv.

[12] Kaazempur-Mofrad MR (2004). Characterization of the atherosclerotic carotid bifurcation using MRI, finite element modeling, and histology. Ann Biomed Eng.

[13] Kimura BJ (1996). Atheroma morphology and distribution in proximal left anterior descending coronary artery: *in vivo* observations. J Am Coll Cardiol.

[14] Konishi T, Yamamoto T, Funayama N, Nishihara H, Hotta D (2016). Relationship between left coronary artery bifurcation angle and restenosis after stenting of the proximal left anterior descending artery. Coron Artery Dis.

[15] Pflederer T, Ludwig J, Ropers D, Daniel WG, Achenbach S (2006). Measurement of coronary artery bifurcation angles by multidetector computed tomography. Invest Radiol.

[16] Wentzel JJ (2012). Endothelial shear stress in the evolution of coronary atherosclerotic plaque and vascular remodelling: current understanding and remaining questions. Cardiovasc Res.

[17] Sun Z, Xu L, Fan Z (2016). Coronary CT angiography in calcified coronary plaques: Comparison of diagnostic accuracy between bifurcation angle measurement and coronary lumen assessment for diagnosing significant coronary stenosis. Int J Cardiol.

[18] Rodriguez-Granillo GA (2006). Plaque composition and its relationship with acknowledged shear stress patterns in coronary arteries. J Am Coll Cardiol.

[19] Juan YH (2014). The evolution and investigation of native coronary arteries in patients after coronary stent implantation: a study by 320-detector CT angiography. Int J Cardiovasc Imaging.

[20] Chen CC (2011). The effect of calcium score on the diagnostic accuracy of coronary computed tomography angiography. Int J Cardiovasc Imaging.

[21] Hong MK (2005). The site of plaque rupture in native coronary arteries: a three-vessel intravascular ultrasound analysis. J Am Coll Cardiol.

[22] von Birgelen C (2001). Plaque distribution and vascular remodeling of ruptured and nonruptured coronary plaques in the same vessel: an intravascular ultrasound study *in vivo*. J Am Coll Cardiol.

[23] Bazzocchi G, Romagnoli A, Sperandio M, Simonetti G (2011). Evaluation with 64-slice CT of the prevalence of coronary artery variants and congenital anomalies: a retrospective study of 3,236 patients. Radiol Med.

[24] O′Brien JP, Srichai MB, Hecht EM, Kim DC, Jacobs JE (2007). Anatomy of the heart at multidetector CT: what the radiologist needs to know. Radiographic.

[25] Kosar P, Ergun E, Ozturk C, Kosar U (2009). Anatomic variations and anomalies of the coronary arteries: 64-slice CT angiographic appearance. Diagn Interv Radiol.

